# A glycoengineered therapeutic anti-HBV antibody that allows increased HBsAg immunoclearance improves HBV suppression *in vivo*

**DOI:** 10.3389/fphar.2023.1213726

**Published:** 2023-12-27

**Authors:** Min You, Fentian Chen, Chao Yu, Yuanzhi Chen, Yue Wang, Xue Liu, Xueran Guo, Bing Zhou, Xin Wang, Boya Zhang, Mujin Fang, Tianying Zhang, Ping Yue, Yingbin Wang, Quan Yuan, Wenxin Luo

**Affiliations:** ^1^ State Key Laboratory of Molecular Vaccinology and Molecular Diagnostics, School of Public Health, School of Life Science, National Institute of Diagnostics and Vaccine Development in Infectious Diseases, Xiamen University, Xiamen, China; ^2^ The 2nd Affiliated Hospital, South University of Science and Technology, Shenzhen, China; ^3^ Xiang An Biomedicine Laboratory, Xiamen, China; ^4^ School of Biology and Engineering (School of Health Medicine Modern Industry), Immune Cells and Antibody Engineering Research Center in University of Guizhou Province, Guizhou Medical University, Guiyang, China

**Keywords:** HBsAg, chronic hepatitis B infection, therapeutic antibody, phagocytosis, neutralization, defucosylation

## Abstract

**Introduction:** The effective and persistent suppression of hepatitis B surface antigen (HBsAg) in patients with chronic HBV infection (CHB) is considered to be a promising approach to achieve a functional cure of hepatitis B. In our previous study, we found that the antibody E6F6 can clear HBsAg through FcγR-mediated phagocytosis, and its humanized form (huE6F6 antibody) is expected to be a new tool for the treatment of CHB. Previous studies have shown that the glycosylation of Fc segments affects the binding of antibodies to FcγR and thus affects the biological activity of antibodies *in vivo*.

**Methods:** To further improve the therapeutic potential of huE6F6, in this study, we defucosylated huE6F6 (huE6F6-fuc-), preliminarily explored the developability of this molecule, and studied the therapeutic potential of this molecule and its underlying mechanism *in vitro* and *in vivo* models.

**Results:** huE6F6-fuc- has desirable physicochemical properties. Compared with huE6F6-wt, huE6F6-fuc- administration resulted in a stronger viral clearance *in vivo*. Meanwhile, huE6F6-fuc- keep a similar neutralization activity and binding activity to huE6F6-wt *in vitro*. Immunological analyses suggested that huE6F6-fuc- exhibited enhanced binding to hCD32b and hCD16b, which mainly contributed to its enhanced therapeutic activity *in vivo*.

**Conclusions:** In summary, the huE6F6-fuc- molecule that was developed in this study, which has desirable developability, can clear HBsAg more efficiently *in vivo*, providing a promising treatment for CHB patients. Our study provides new guidance for antibody engineering in other disease fields.

## 1 Introduction

Hepatitis B virus (HBV) is one of the most important pathogens in the world and may cause chronic or even life-long infections (Organization, 2021). Chronic HBV infection (CHB) causes chronic hepatitis and results in high risk of death due to cirrhosis and hepatocellular carcinoma (HCC) (Kremsdorf et al., 2006; Dienstag, 2008; Wu et al., 2021). Globally, approximately 820,000 people die from acute or chronic HBV infection in 2019 (Jeng et al., 2023). HBV infection has become a global public health problem. The successful production of a preventive hepatitis B vaccine has effectively reduced the number of new cases of HBV infection worldwide; however, hundreds of millions of people remain infected with HBV, and some of them need effective therapy to prevent the disease from getting worse (Nayagam et al., 2016; Organization, 2021). The anti-HBV drugs that have approved for marketing, namely, interferon or nucleos(t)ide analogs, can only alleviate the disease but cannot eradicate the virus or completely block HCC development (Kwon and Lok, 2011; Fanning et al., 2019; Shen et al., 2020). The ideal endpoint of anti-HBV therapy, which is also called the functional cure of hepatitis B infection, is defined by the loss of hepatitis B surface antigen (HBsAg) expression. However, currently available drugs rarely achieve this goal; in fact, less than 5% of cases are cured by these treatments (European Association For The Study Of The L, 2012; Trepo et al., 2014; Hou et al., 2020). There is an urgent need to develop more effective anti-HBV drugs or therapeutic strategies (Zoulim, 2007).

Antibody drugs have been successfully applied in the treatment of various diseases, such as cancer, autoimmune diseases and viral infection, due to their excellent targeting specificity and safety (Carter and Lazar, 2018; Zhang et al., 2018). In the field of respiratory syncytial virus treatment, the neutralizing monoclonal antibody Synagis leads to rapid and profound virus clearance in patients (Resch, 2017; Tang, 2017; Wong et al., 2018). REGEN-COV™ (casirivimab and imbdevimab) is an antibody cocktail that is used to treat Corona Virus Disease 2019 (Baum et al., 2020). A phase 3 trial showed that treatment with REGEN-COV significantly reduced the risk of hospitalization or death, and it has a safety profile that is consistent with previously reported data (Weinreich et al., 2021). The FDA issued an emergency use authorization (EUA) for REGEN-COV™ (which is produced by Regeneron, (2021)) to treat mild-to-moderate COVID-19 in adults and children over the age of 12 in 2020. A lower 1,200 mg intravenous and subcutaneous dose of REGEN-COV™ was approved by the FDA to treat patients with COVID-19 (2021). Recent research on the treatment of CHB infection has reported that antibodies that recognize three epitopes can rapidly and efficiently clear viruses in mouse models (Wang et al., 2016; Zhang et al., 2016; Li et al., 2017). Injection of virus-specific antibodies may achieve viral clearance through multiple mechanisms (Marasco and Sui, 2007). Antibodies, the CDR regions of which target the viral envelope, block virus entry into host cells. As a result, further infection is prevented (Marasco and Sui, 2007). In addition, the Fc regions of antibodies may mediate, for example, virus antigen clearance or virus-producing cell phagocytosis, antibody-dependent cell-mediated cytotoxicity, complement-mediated lysis or humoral tolerance in the body to directly eliminate circulating virus (Marasco and Sui, 2007; Nimmerjahn and Ravetch, 2008; Delidakis et al., 2022).

Similar to other secreted glycoproteins, IgGs are glycosylated in the ER and Golgi in mammalian cells. N279, which is located in the CH2 domain of each heavy chain of human IgG1, is an N-glycosylation site, and specific glycans are added to N279, resulting in multiple glycoforms. Although a particular IgG subtype Fc region has a highly conserved primary sequence, diverse N-glycosylation patterns have been shown to alter the conformations and even the functions of IgG molecules (Chen et al., 2017; Goletz et al., 2018). Several successful drug development studies have shown that glycosylation engineering of the Fc segments of therapeutic antibodies, such as benralizumab (Laviolette et al., 2013) and mogamulizumab (Kanazawa et al., 2014; Remer et al., 2014), can effectively improve treatment.

In this study, we explored the feasibility of glycosylation engineering of an HBV mAb as a treatment for HBV infection. A glycoengineered antibody targeting HBV (huE6F6-fuc-) was prepared by expressing an HBV-specific monoclonal antibody (huE6F6) in *FUT8*-deficient CHO cells. Moreover, the physicochemical properties of the glycoengineered huE6F6 were characterized, and the therapeutic effects of huE6F6-fuc- and the underlying mechanism were systematically investigated. This will provide direct evidence that will further promote the development of this drug for marketing.

## 2 Materials and methods

### 2.1 Cell line

The CHO-9618s cell line is derived from CHO K1 cells (ATCC, CRL-9618), which were obtained from American Type Culture Collection, and it was adapted for suspension culture in serum-free medium. CHO-9618s cells were cultured in SFM IV medium (HyClone, SH30518.03) with 8 mM L-Glutamine (SIGMA-ALDRICH, G8540).

### 2.2 Gene editing

In the CHO genome, the FUT8 gene is composed of 9 exons (Supplementary Figure S1A). The sequence of FUT8 in the CHO genome is highly homologous with the human FUT8 gene (Supplementary Figure S1B); thus, it is hypothesized that the structure of CHO FUT8 is similar to that of human FUT8. According to the crystal structure of human FUT8 (Gene ID: 100751648; Protein-id: xp_007640580.1), exon 7 of Fut8 encodes the substrate-binding region of the enzyme (Supplementary Figure S1A–C).

The glutamine synthetase (GS) gene, which contains six exons (Supplementary Figure S2A), is a highly conserved gene in evolution. The homology of the CHO GS protein and the human GS protein is as high as 94% (Supplementary Figure S2B). Because the structure of the GS protein from CHO cells has not yet been released, this study referred to human GS protein (Gene ID: 100689337; Protein-id: 001233699.1). The monomer GS protein polymerizes into a five-polymer structure, and further polymerizes into a 10-polymer structure (Supplementary Figure S2C). Exon 5 of GS, which is located on the 5-polymer interface, is important for maintaining the structure and function of the protein.

To knock out FUT8, we designed two sgRNAs (Small guide RNA) (FUT8 7-1 and FUT8 7–2) that target exon 7 of the FUT8 gene (Supplementary Figure S1D). Two sgRNAs (Exons 5-5 and 5–3) were synthesized that target the exon 5 flanks of the GS gene. Moreover, the plasmid, which was named TARGET and was homologous to intron 4 and intron 5 of the GS gene, was constructed, and the selection marker zeocin was inserted between intron 4 and intron 5 (Supplementary Figure S2D). It was expected that the zeocin gene would homologically replace exon 5 of GS.

The PX330 plasmids carrying the sgRNAs and the TARGET plasmid were cotransfected into CHO-9618s cells and screened by being cultured with lectin and zeocin. The cells that could resist lectin and zeocin treatment were cloned from a single cell.

### 2.3 Cell surface staining

The cells in the logarithmic growth phase were collected by centrifugation at 1000 rpm for 5 min, washed with PBS twice, and stained with Lac-FITC (Vector Laboratories, L-1040–25) at room temperature for 20 min. After washing with PBS 2 times, the stained cells were analyzed by flow cytometry and inverted fluorescence microscopy.

### 2.4 L-Glutamine (Lg)-dependent test

Cells were seeded in medium with or without Lg at 1E5 c/mL, and the number of cells was counted after being cultured for 1 week.

### 2.5 Sequence of the edited gene

Whole-cell genome extraction was performed according to the operation manual of the Qiagen Cell Genome Extraction Kit. The fragments of GS-exon 5 and FUT8-exon 7 were obtained by PCR and were subjected to sequencing.

### 2.6 Cell culture with fed-batch

Cells were seeded in SFM IV medium at 0.5E6 c/mL and fed Feed C (6%, 10%, 8%, 6% and 6%) on days 3, 6, 8, 10 and 12. The concentration of glucose was maintained at 5 g/L, and the culture temperature was decreased to 32°C on day 6. The cultures were harvested on day 14. The cell density and viability were measured daily during culture.

### 2.7 Transient expression

The mAb genes were cloned into a recombinant mammalian expression vector and expressed in CHO-FGKO or CHO-WT cells. The method was described previously (You et al., 2013).

### 2.8 Expression in stable cell lines

The antibody genes were inserted into the pGS2 plasmid. A stable cell pool was developed as described previously (You et al., 2018). The top mini pool was cultured with fed-batch as described in 2.1.6. The harvested protein was purified with protein A.

### 2.9 SDS‒PAGE

The purified antibody was suspended in nonreduced loading buffer or reduced loading buffer, and it was electrophoresed in a 10% SDS‒PAGE gel. After electrophoresis, the gel was stained with Coomassie for 1.5–2.0 h and then decolorized with ultrapure water until the bands were clearly visible. Finally, the gel was imaged.

### 2.10 Size-exclusion chromatography-high-performance liquid chromatography (SEC-HPLC)

The assay was performed as described previously (You et al., 2018).

### 2.11 Differential scanning calorimetry

Differential scanning calorimetry (DSC) was performed with a MicroCal VP-DSC instrument (GE Healthcare, VP-Capillary DSC). All the samples were diluted to 0.8 mg/mL and heated from 10°C to 90°C at a constant heating rate of 1°C per minute. The melting temperatures (Tm) were calculated by MicroCal Origin 8.0 (Origin-Lab Corp.) software.

### 2.12 CE-SDS analysis

CE-SDS analysis was conducted with a AB Sciex PA800 plus pharmaceutical analysis system and the SDS-MW Analysis Kit, which is designed for the separation of protein-SDS complexes using a replaceable gel matrix. The gel is formulated to provide an effective sieving range of approximately 10 kDa–225 kDa. For reduced samples, 95 μL of 0.5 mg/mL antibody was mixed with 5 μL of 2-mercaptoethanol and 2 μL of internal standard, and the mixture was heated in a water bath at 100°C for 3 minutes. For nonreduced samples, the 2-mercaptoethanol was replaced with iodoacetamide solution, and the mixture was heated in a water bath at 70°C for 3 minutes. The samples were then analyzed according to the operation guide of the SDS-MW Analysis of PA 800 plus Pharmaceutical Analysis System. A Size Standard was used to estimate the protein molecular weight of the sample.

### 2.13 Capillary Isoelectric Focusing (cIEF) analysis

Capillary Isoelectric Focusing (cIEF) Analysis was performed by AB PA800 plus pharmaceutical analysis systems, and a commercial CIEF kit (Beckman, A80976) was used according to the manufacturer’s instructions. In brief, cIEF separation consists of two steps, namely, focusing and mobilization, and the cIEF sample includes a mixture of ampholytes, stabilizers, pI markers, and the protein to be detected. Ten microliters of huE6F6 (8 mg/mL) was mixed with the following reagents in a centrifuge tube: 200 μL of 3 M urea-cIEF gel, 12 μL of Pharmalyte 3–10 carrier ampholytes, 20 μL of cathodic stabilizer, 2 μL of anodic stabilizer, and 2 μL of each pI marker (pI = 10.0/9.5/5.5). Finally, detection in cIEF was performed at 280 nm with a UV detector.

### 2.14 N-glycosylation analysis

In this study, the release of N-glycan from antibodies was analyzed by a Beckman PA800 plus pharmaceutical analysis system. A commercial Carbohydrate Labeling & Analysis Kit (Cat# 477600) was used in this assay. This kit contains a Carbohydrate Separation Buffer, N-CHO Coated Capillary, Labeling Dye (APTS), Labeling Dye Solvent and Glucose Ladder Standard. Briefly, the oligosaccharides were first released (PNGase F, New England Biolabs, P0704S)) and purified from 200 μg huE6F6. After release, the oligosaccharides were labeled with a fluorophore called 1-aminopyrene-3,6,8-trisulfonic acid (APTS) as a guide. Finally, analysis of the fluorophore-labeled oligosaccharides was performed by N-CHO capillary-based capillary electrophoresis, and the fluorescence was measured by an LIF detector and analyzed by 32 Karat (Version 10.1). A 50 μm I.D. N-CHO capillary with a 50.2 cm total length (40.0 cm effective length) was installed into the capillary cartridge.

### 2.15 The binding to HBsAg or peptides

The antibodies were serially diluted at 1:2 using a workstation (Beckman, NXP) beginning at 1000 ng/mL. The diluted samples in three replications were added to 96-well plates coated with HBsAg or peptide, and the binding activity was investigated by a chemical luminescence assay (CLA). The CLA values were input into Prism 7 for EC50 calculation.

### 2.16 Competition ELISA

An additional preincubation with a 10-fold molar excess of unlabeled competitor antibodies was performed before adding the HRP-labeled mAbs to the HBsAg-coated plates. Competition levels were calculated as the percent inhibition of the half-maximal binding concentration of the test antibody relative to the absorbance without added competitor (Shen et al., 2019).

### 2.17 Neutralization assay *in vitro*

HBV that was produced by HepaAD38 cells and serial dilutions of huE6F6 were incubated for 1 h at 37°C. Then, the mixtures were added to NTCP-expressing HepG2 cells. The levels of HbsAg that were secreted into the culture supernatants were measured on the seventh day post infection. The procedures were previously described (Zhang et al., 2020). We used HBIG as the positive control, and its blocking rate was used as the standard curve. The inhibition values were input into Prism 7 for ED50 calculation.

### 2.18 Treatment of HBV infection in mice

HBV-transgenic (HBV-Tg) mice were provided by Pei-Jer Chen (NTU, Taiwan). For *in vivo* treatment of the mice with mAbs, 200 ug mAb in PBS was injected into the mice through the tail vein. The mice were bled at baseline and at the indicated time points after mAb infusion. Mouse serum samples were stored at −80°C until use. Quantification of the levels HBsAg and HBV DNA in the serum samples was conducted as previously described (Wang et al., 2022). The serum titer of huE6F6 was determined by a quantitative anti-human antibody ELISA kit. The pharmacokinetic parameters of huE6F6-wt and huE6F6-fuc- in mice were calculated using DAS ver 2.0.

### 2.19 Analysis of the antibody-mediated internalization of HBsAg by differentiated THP-1 cells

The antibody was serially diluted with RPMI +10% FBS, with an initial concentration of 20 μg/mL. The diluted antibody was mixed with an equal volume of 800 ng/mL HBsAg. The mixture was incubated at 37°C for 1 h. Then, the HBsAg antibody samples were added to differentiated THP-1 cells at a density of 2E6 cells/mL. The HBsAg-antibody-THP-1 mixtures were coincubated at 37°C. After 2 hours of incubation, the cells were washed with 500 μL of PBS once and exposed to 120 µL of DDM lysate at 4°C for 1 h. Finally, 100 µL of lysate was removed for the quantitative analysis of the HBsAg levels.

### 2.20 *In vitro* THP-1 cell opsonophagocytosis analysis by FAC

DyLight 488 (Thermo Scientific, 46402)-labeled HBsAg (HBsAg-DyLight 488) was mixed with antibody at a ratio of 1:1 in RPMI 1640 + 10% FBS and incubated at 37°C for 1 h. The cell density of differentiated THP-1 cells was adjusted to 5E5 cells/mL, and then, the cells were added to the HBsAg-DyLight 488-Antibody mixture. After 2 hours of incubation at 37°C, the samples were washed once with 500 μL of PBS, resuspended in 500 μL of PBS and stored at 4°C for flow cytometric analyses using a FACSAriaTM III (BD Biosciences, Facsaria III). The data were analyzed using the FlowJo 7.6 software package.

### 2.21 *In vitro* opsonophagocytosis by PBMCs

The study protocol was reviewed and approved by the internal ethical committee of the school of medicine, Xiamen University, China. Written informed consent was obtained from each healthy adult volunteer according to the principles of the Declaration of Helsinki, and then 5 mL peripheral blood samples were collected.

A total of 2000 IU HBsAg was mixed with antibody at 20 ug/mL and incubated at 37°C for 1 h. Human peripheral blood cells (PBMCs) from three healthy donors were added to the mixture and used as effector cells. After incubation for 6 h at 37°C, the supernatants were collected by centrifugation and analyzed with a quantitative HBsAg kit (Wantai, Beijing, HBV-1396). Phagocytosis percentages = 100% Ⅹ (Initial concentration of HBsAg—HBsAg concentration in supernatants post incubation with antibody and effector cells)/Initial concentration of HBsAg.

### 2.22 Serum cytokine profiles

The serum cytokine profiles were assayed according to the Luminex-based MILLIPLEX MAP.

Mouse Cytokine/Chemokine Magnetic Bead Panel and the Immunology Multiplex Assay Kit (Millipore, MCYTOMAG-70K).

### 2.23 Measurement of antibody binding to FcγRs by biolayer interferometry (BLI)

Kinetic analysis of the binding of huE6F6-wt or huE6F6-fuc- to human FcγRs or mouse FcγRs was performed using the Fortebio Octet system at 30°C. The assay was performed using NTA sensors in which FcγRs were immobilized onto binding sensor tips in assay buffer, which consisted of 1× PBS, 0.1% BSA, and 0.02% Tween 20. FcγR was loaded onto NTA biosensors (ForteBio, 18–5101) at 5 μg/mL to yield a response of 2.5 nm. During the association step, six 2-fold serial dilutions of the antibodies, with the highest concentration at ∼10×KD (known or predicted), along with buffer-only wells, was used. The association phase lasted for 60 s, and the dissociation phase lasted for 60 s followed by a regeneration step with 10 mM glycine-HCl, pH 1.7. The data were analyzed using the Octet data analysis program. Steady-state equilibrium analysis was used to estimate binding affinities.

### 2.24 *In vivo* tracking of antibody distributions by fluorescence imaging

The antibodies were labeled with Cy5.5 to generate huE6F6-wt-Cy5.5 and huE6F6-fuc-Cy5.5. SPF mice were intravenously injected with huE6F6-wt-Cy5.5 or huE6F6-fuc-Cy5.5 (5 mg/kg). Twenty-4 hours after administration, the fluorescence signal of Cy5.5 in mice was recorded by an IVIS Lumina II system (Caliper Life Sciences, United States of America) under anesthesia. Then, the animals were sacrificed, and the major viscera (heart, liver, spleen, lung, and kidney) were isolated and subjected to fluorescence analysis. All the animal experiments were conducted in accordance with the *Guide for the Care and Use of Laboratory Animals*.

### 2.25 Characterization of the mAb-antigen immune complex

The morphologies of the antibody-virion ICs induced by huE6F6-wt or huE6F6-fuc- were investigated using TEM. HBsAg was obtained by recombinant expression. HBsAg (0.4 mg/mL) was incubated with mAbs (0.8 mg/mL) at 37°C for 60 min to induce antibody-antigen immune complex formation and then negatively stained for TEM observation using FEI Tecnai G2 Spirit BioTWIN (FEI, Hillsboro, OR).

## 3 Results

### 3.1 Development of FUT8-and GS-knockout CHO cells

**FIGURE 1:**
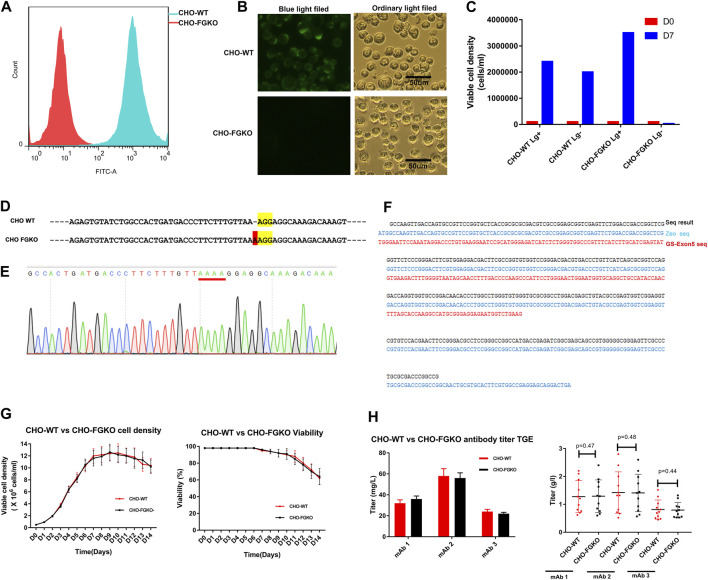
Development of *FUT8−/−GS−/−* knockout CHO cells. **(A)** CHO-WT and CHO-FGKO cells stained with lectin- FITC were analyzed by FACS. **(B)** CHO-WT and CHO-FGKO cells were stained with Lectin-FITC. **(C)** CHO-WT and CHO-FGKO cells were cultured with or without Lg. **(D)** Exon 7 of *FUT8* in CHO-WT and CHO-FGKO were aligned. The nucleotides in yellow are PAM sites, and the nucleotides in red are insertions. **(E)** Sanger sequencing of exon 7 of CHO-FGKO cells is shown. **(F)** *GS* Exon 5 of CHO-FGKO cells was sequenced and assayed. **(G)** CHO-WT and CHO-FGKO cells were cultured with a fed-batch approach. The cell density is shown on the left, and the cell viability is shown on the right. **(H)** CHO-WT and CHO-FGKO cells were used for the expression of antibodies. The left figure shows the transient expression yield, and the stable cell pool expression yield of the three antibodies (mAb1, mAb2 and mAb3) is shown on the right.

In CHO, α-1,6-fucosyltransferase, which is encoded by *FUT8*, is mainly responsible for transferring α(1,6)-linked fucose to the N-glycosylation sites of proteins. Knockout of *FUT8* can effectively remove the fucosylation of proteins (Yamane-Ohnuki et al., 2004). CHO cells that are deficient in GS have obvious advantages for generating stable cell lines and have been successfully used in the industrial production of a variety of commercial antibody drugs (Rajendra et al., 2015). In this study, the *GS* gene and *FUT8* gene were simultaneously knocked out in CHO cells with CRISPR/Cas9 technology and homologous replacement. Finally, a CHO *FUT8* ^*−/−*^ *GS*−/− line, named CHO-FGKO, was cloned. Fluorescence microscopy showed that the cells could not be stained by Lectin-FITC, and similar results were obtained by flow cytometry (Figures 1A, B). We found that the cells could not grow in medium without Lg, indicating that the cells were Lg dependent (Figure 1C).

The sequencing of the *FUT8* gene showed that a nucleotide insertion occurred in exon 7 (Figure 1D), resulting in a frameshift mutation that led to a structural change in the α-1,6-fucosyltransferase protein and the loss of catalytic activity. The sequence of the exon 5 segment of the GS gene showed that the segment had been replaced by the exogenous zeocin gene (Figures 1F, G), resulting in structural collapse of the GS gene and the loss of endogenous glutamine synthase activity. Two pairs of FUT8 alleles and two pairs of GS alleles were simultaneously knocked out, as shown by whole-genome sequencing (results not shown).

To explore whether CHO-FGKO cells could be used in large-scale antibody production, the growth characteristics and expression levels of CHO-FGKO were investigated. The parental CHO (CHO-WT) and CHO-FGKO cell lines were seeded at 0.5E6 c/mL and cultured with a fed-batch process. As shown in Figure 1G, the cell density peaked at approximately Day 10, and the peak density were 12.25E6 cells/mL and 11.6E6 cells/mL respectively. The viability began to decline on day 6. At the end of culture, the cell density decreased to 64%. In general, CHO-FGKO cells had the same proliferation characteristics as CHO-WT cells.

Genes encoding three antibodies (mAb1, mAb2 and mAb3) were constructed into expression vectors and transfected into CHO-FGKO and CHO-WT cells by transient transfection. For the three antibodies, the expression levels in the two host cells were almost identical (Figure 1H). Using CHO-FGKO or CHO-WT cells as hosts, stable cell lines that expressed the three antibodies (mAb1, mAb2, and mAb3) were developed as previously described (You et al., 2018). The top 10 pools of each antibody were subjected to fed-batch culture, and the supernatants were analyzed at the end of the culture. For mAb1, the average yield of the pools derived from CHO-WT cells was 1.28 g/L, while the pools derived from CHO-FGKO cells gave an average yield of 1.29 g/L. The expression levels of mAb2 in CHO-WT and CHO-FGKO cells were 1.42 and 1.41 g/L, respectively. The yields of mAb3 in CHO-WT and CHO-FGKO cells were 0.81 and 0.79 g/L, respectively. This indicates that there is no significant difference in cell growth and production potential between CHO-WT and CHO-FGKO cells (*p* > 0.1), and CHO-FGKO cells could be applicable for the large-scale production of antibody drugs.

In this part, *FUT8* and *GS* were knocked out in CHO cells by CRISPR technology and homologous recombination. The growth and expression level of CHO-FGKO cells were almost the same as those of parental CHO cells.

### 3.2 Physicochemical properties of huE6F6-wt and huE6F6-fuc-

E6F6 is the mouse monoclonal antibody, which was subject to CDR-grafting and structure guided maturation at CDR region. Finally, the humanized antibodies huE6F6 in this study were obtained. huE6F6 is a potential therapeutic antibody for HBV(Zhou et al., 2020). Wild-type huE6F6 (huE6F6-wt) was expressed in CHO-WT (CHO-9618s) cells, and defucosylated huE6F6 (huE6F6-fuc-) was produced by CHO-FGKO cells. huE6F6-wt and huE6F6-fuc- were purified with Protein A affinity chromatography, and the physicochemical properties were analyzed by methods including SDS‒PAGE, SEC-HPLC, CE-SDS, CIEF, DSC and N-glycosylation.

**FIGURE 2:**
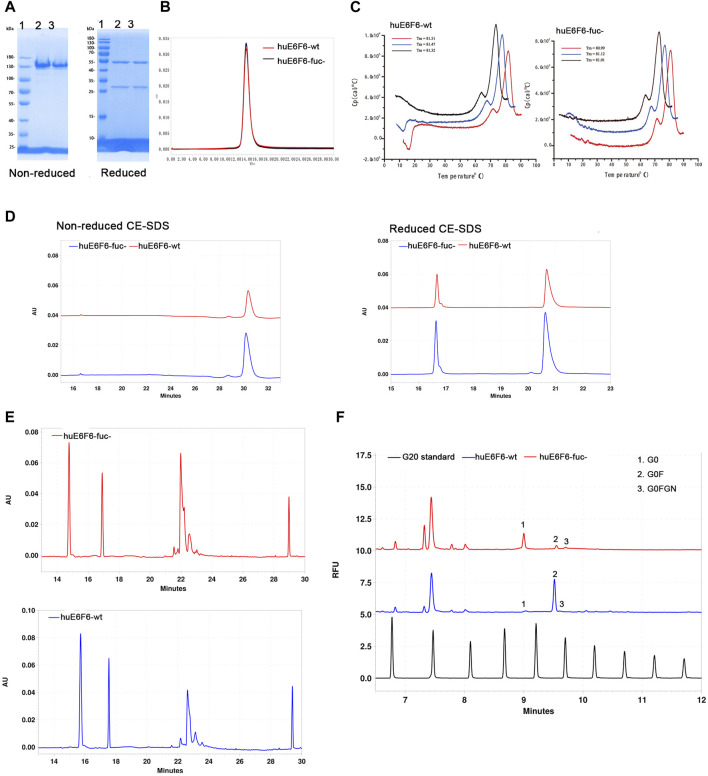
Physicochemical properties of huE6F6-wt and huE6F6-fuc-. **(A)** SDS‒PAGE analysis of the antibodies under nonreduced or reduced conditions. Lane 1, protein markers; lane 2, huE6F6-wt; lane 3, huE6F6-fuc-. **(B)** SEC assay of huE6F6-wt and huE6F6-fuc-. **(C)** DSC assay of huE6F6-wt and huE6F6-fuc- in PBS. **(D)** CE-SDS analysis of huE6F6-wt and huE6F6-fuc- under nonreduced or reduced conditions. **(E)** The ciEF assay of huE6F6-wt and huE6F6-fuc-. **(F)** The N-Glycan assay of huE6F6-wt and huE6F6-fuc- with PA800.

The same amounts of huE6F6-wt and huE6F6-fuc- were assayed with reduced and nonreduced SDS‒PAGE (Figure 2A). In nonreduced SDS‒PAGE, both huE6F6-wt and huE6F6-fuc- exhibited molecular weights of 150 Kd. In reduced SDS‒PAGE, a band of 50 kDa and a band of 25 kDa, which represent the heavy and light chain respectively, were observed.

SEC-HPLC analysis was performed with huE6F6-wt and huE6F6-fuc- (Figure 2B). The retention time of huE6F6-wt and huE6F6-fuc- was 13.8 min, and the main peak areas were 99.2% and 99.3%, respectively. According to SDS‒PAGE and SEC-HPLC analysis, the difference between the two antibodies was not significant. The main reason is that the resolution of these two methods is not sufficient to separate the differences in the glycans on huE6F6-wt and huE6F6-fuc-.

To study the effect of defucosylation on the thermal stability of the huE6F6 antibody, differential scanning calorimetry (DSC) was used to analyze the thermal melting temperatures (T_m_). As shown in Figure 2C, the Tm1 of huE6F6-wt was 71.46°C while the Tm1 of huE6F6-fuc- was 71.65°C. The Tm2 of huE6F6-wt was 81.43°C, while the Tm2 of huE6F6-fuc- was 81.04°C. This result suggests that the thermal stability of huE6F6-fuc- decreased slightly due to defucosylation. This phenomenon is consistent with Ryuta’s study (Wada et al., 2018). According to the Tm value of these antibody drugs, the Tm of huE6F6-fuc- was high enough to support drug development (Supplementary Table S1).

CE-SDS analysis was carried out on huE6F6-wt and huE6F6-fuc-. In nonreduced CE-SDS, the retention time of huE6F6-wt was 30.18 min, and the retention time of huE6F6-fuc- was 30.3 min, which was slightly longer than that of huE6F6-wt (Figure 2D). The results showed that the absence of fucosylcan caused a slight increase in retention time. The antibodies were separated into their light chain and heavy chain fractions by reduced CE-SDS analysis. For huE6F6-wt, the retention time of the light chain (peak 1) was 16.64 min, and the retention time of the heavy chain (peak 2) was 20.70 min. The retention time of the light chain peak (peak 1) of huE6F6-fuc- was 16.64 min, and the retention time of the heavy chain peak (peak 2) was 20.62 min. The retention times of the light chains of huE6F6-wt and huE6F6-fuc- were basically identical, while the retention time of the heavy chain peak of huE6F6-wt was slightly longer than that of huE6F6-fuc-.

The charge of an antibody may affect its metabolism *in vivo*. Therefore, CIEF was used to analyze the charge variants of huE6F6. As shown in Figure 2E, huE6F6-wt mainly contained five variants, the pI ranged from 7.4 to 8.1, and the pI of the main variant (peak 3, at 22.63 min) was 7.8. However, huE6F6-fuc- mainly contained four variants, with a pI range of 7.4–7.9, and the pI of the main variant (peak 3, at 22.1 min) was 7.7. It is suggested that the absence of fucosyltransferase increased acidic variants.

To investigate the N-glycoform of huE6F6, the glycans were released from huE6F6 and subjected to CE-SDS analysis (Figure 2F). The main glycoform of huE6F6-wt was G0F (98%), while the major glycoform of huE6F6-fuc- was G0 (85%). In this study, only Protein A affinity chromatography was used to purify the antibody, and variants (peak 2 and peak 3) with molecular weights similar to those of G0FGN and G0F may represent immature antibodies in huE6F6. In industrial production, three affinity chromatography methods, including one protein A column, anion exchange and cation exchange, are usually carried out. We believe that the purity of the G0 glycoform can exceed 98% after three-step purification.

In this part, we preliminarily analyzed the physicochemical properties of huE6F6-fuc-. According to SDS‒PAGE and SEC-HPLC analysis, huE6F6-fuc- was basically the same as huE6F6-wt. According to higher resolution methods of analysis, such as CE-SDS‒PAGE and CIEF, the two antibodies had some differences. The Tm of huE6F6-wt was slightly lower than that of huE6F6-fuc-, which may be caused by the absence of fucosylcan. The detection of cIEF showed that the pI of huE6F6-wt was 7.8 and that of huE6F6-fuc- was 7.7–7.8. In general, huE6F6-fuc- was characterized by homogeneous molecules, a low tendency of aggregation and degradation, and good thermal stability, which could support further drug development.

### 3.3 Binding and neutralizing activity of antibodies *in vitro*

**FIGURE 3:**
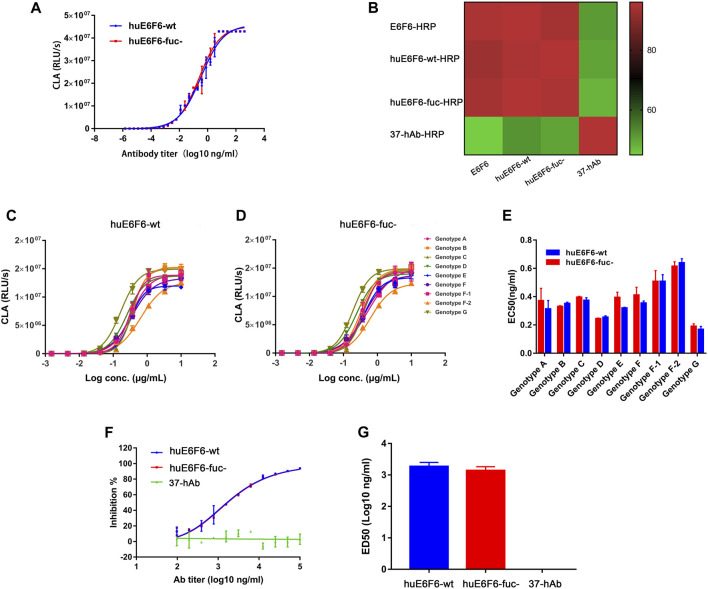
Binding and neutralizing activity of antibodies *in vitro*. **(A)** The binding activity of huE6F6-wt and huE6F6-fuc- mAbs with HBsAg. **(B)** Competition between antibodies. The competition level is shown in color. E6F6 is the parent molecule of huE6F6-wt and huE6F6-fuc-. 37-hAb, which is a humanized antibody against H5N1, was used as an isotype control. **(C)** Binding of huE6F6-wt to peptide epitopes from all HBV genotypes. **(D)** Binding of huE6F6-fuc- to peptide epitopes from all HBV genotypes. **(E)** The EC50 of huE6F6-wt or huE6F6-fuc- binding the epitope of the different HBV genotypes. **(F)** The neutralization activity of huE6F6-wt and huE6F6-fuc- mAbs against HBV. **(G)** The ED50s were calculated according to Figure 3F.

To explore whether defucosylation would affect the biological function of the huE6F6 antibody, the binding activity and neutralization activity were tested. The ability of huE6F6-wt or huE6F6-fuc- to bind to HBsAg-coated 96-well plates was assayed with CLA, and the results are shown in Figure 3A. The binding curves of HBsAg with huE6F6-wt or huE6F6-fuc- almost coincided. The EC50 of huE6F6-wt with HBsAg was 0.143, while the EC50 of huE6F6-fuc- with HBsAg was 0.138.

E6F6, which is the parent of huE6F6, huE6F6-wt and huE6F6-fuc- were subjected to competitive ELISA, and the competition levels were calculated. As shown in Figure 3B, the mutual competition levels among E6F6, huE6F6-wt and huE6F6-fuc- reached greater than 95%, indicating that the three antibodies can completely block each other and suggesting that the three antibodies recognize the same epitope with similar affinity.

HBV is classified into Genotypes A, B, C, D, E, F, f-1, f-2, G and so on. The AA112-134 epitopes of these subtypes vary slightly. The binding of huE6F6 to peptide epitopes of these various subtypes is shown in Figures 3C–E. The trends of the binding curves of the two antibodies to various epitopes was basically the same. The EC50 of the binding of the two antibodies to HBsAg ranged from 0.01 to 0.14, and the strongest binding ability was observed with the g-type epitope peptides. The EC50 of huE6F6-fuc- was 0.012, while the EC50 of huE6F6-wt as 0.011. In comparison, the binding energy of these antibodies to the F2 type was the weakest, and the EC50 of huE6F6-wt was 0.133, while the EC50 of huE6F6-fuc- was 0.142. In general, the two antibodies had basically consistent EC50 values with HBsAg peptide epitopes of each subtype (both *p* values were >0.1).

The *in vitro* infection-neutralizing capabilities of huE6F6-fuc were quantitatively measured, and the results are presented in Figures 3F,G. The blocking effects of the two glycosylated forms of the huE6F6 antibody and the ED50s were found to be almost the same. In summary, these results demonstrated that defucosylation did not affect the binding and neutralizing activities of the huE6F6 antibody.

### 3.4 *In vivo* suppression of HBV by huE6F6-fuc

**FIGURE 4:**
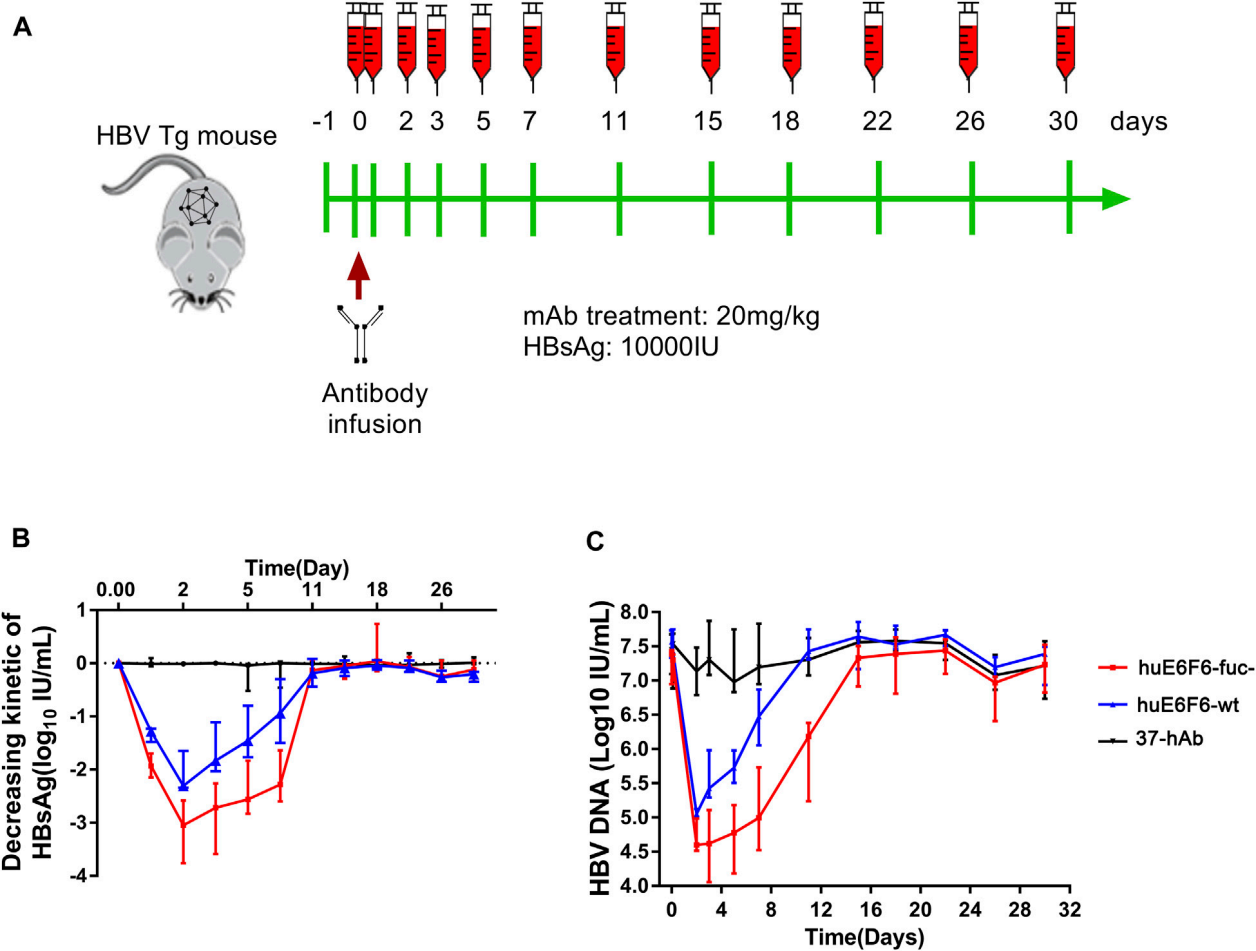
*In vivo* suppression of HBV by huE6F6-fuc-. **(A)** Schematic diagram showing the bleeding and antibody infusion schedules in the mouse study. Five animals were included in each group, except that three were included in the 37-hAb group. **(B)** Decreasing kinetics of HBsAg in HBV-Tg mice after a single mAb treatment. **(C)** HBV DNA concentrations in the mice.

To study the potential of huE6F6-fuc- to be used as a treatment, we evaluated the HBV suppression effect of huE6F6 antibodies on an HBV transgenic mouse model (HBV-Tg). Two groups of HBV-Tg mice that were administered HBsAg at 10000 IU/mL (n = 5) were treated with a single injection of huE6F6-wt or huE6F6-fuc- at a dose of 20 mg/kg. Blood samples were collected at 1 h, 1 ay, 3 days, 5 days, 7 days, 11 days, 15 days, 22 days, 28 days and 30 days post injection (Figure 4A). As shown in Figures 4B,C, treatment with huE6F6-wt or huE6F6-fuc- resulted in a significant decrease in the levels of HBsAg and HBV DNA. After the injection, the HBsAg and HBV-DNA levels decreased rapidly, and the HBsAg levels in the two treatment groups returned to baseline on the 11th day. Within 120 h post injection, the HBsAg and HBV DNA levels in the group that was treated with huE6F6-fuc- were significantly lower than those in the group that was treated with huE6F6-wt (*p* < 0.05). The median level of HBsAg in the group that was treated with huE6F6-fuc- was lower than that in the huE6F6-wt-treated group by 0.5log_10_ at 1 h post injection. At 24 h post treatment, the HBsAg levels in the huE6F6-fuc- group were twice as low as those in the huE6F6-wt group. The HBV DNA levels showed the same trend. Treatment with huE6F6-fuc- neither changed the levels of hepatitis Be antigen (HBeAg) and alanine aminotransferase (ALT) nor induced toxicity in other organs (data not shown). This suggests that the huE6F6-fuc- antibody mediates stronger and more persistent HBV suppression *in vivo*.

We monitored the titer of huE6F6 after a single intravenous injection into HBV-Tg mice, and the PK and PD properties of huE6F6-wt and huE6F6-fuc- were analyzed. The results are shown in Supplementary Figure S3 and Supplementary Table S2. The PK/PD parameters of the two antibodies in mice were very similar. The half-life of huE6F6-wt was 33.544 h, while that of huE6F6-fuc- was 33.559 h. We also studied the distribution of huE6F6-wt or huE6F6-fuc- in mice, and the results are shown in Supplementary Figure S4. The tissue distribution of huE6F6s was basically the same, indicating that defucosylation modification did not change the tissue distribution characteristics of huE6F6.

### 3.5 huE6F6-fuc- clears HBsAg by enhancing antigen phagocytosis

**FIGURE 5:**
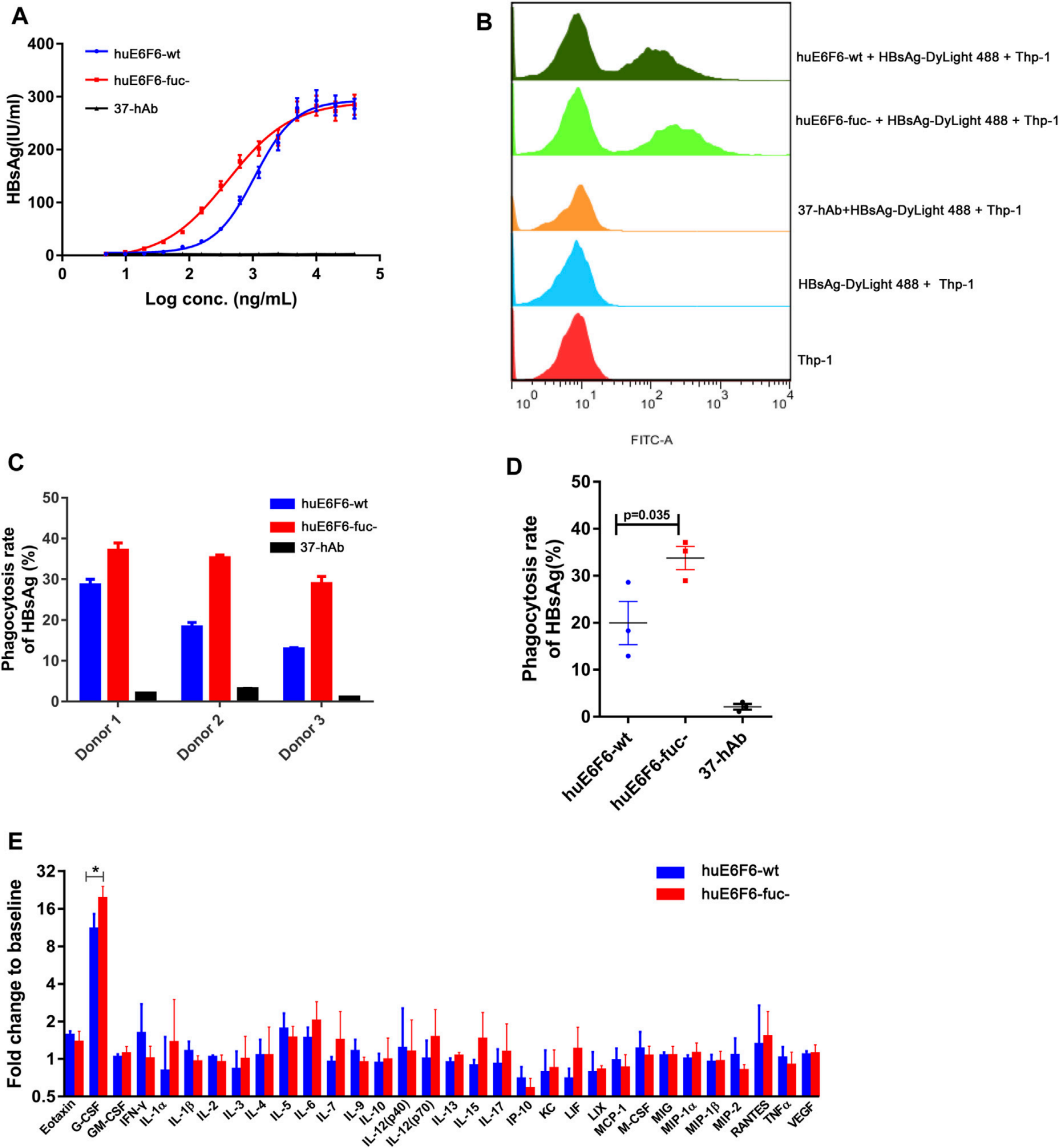
Defucosylation enhances antigen phagocytosis. **(A)** The level of antibody-mediated internalization of HBsAg by differentiated THP-1 cells. **(B)** Flow cytometric analyses of the mAb-HBsAg ICs in differentiated THP-1 cells. **(C)** The huE6F-mediated phagocytosis of HBsAg by PBMCs. **(D)** Scatter plot analysis of phagocytosis efficiency by PBMCs. **(E)** Serum cytokine profile of HBV-Tg mice (n = 3) that received mAbs at 6 h after infusion.

To study the effect of huE6F6 defucosylation on HBsAg phagocytosis, differentiated human THP-1 macrophages and human peripheral blood cells (PBMCs) were used as effector cells *in vitro*. After incubation of the antibodies at different concentrations with HBsAg, we added the mixture to THP-1 cells. HBsAg levels in THP-1 cells was quantified after incubation for 2 h. As shown in Figure 5A, isotype antibody 37-hAb could not mediate the phagocytosis of HBsAg by THP-1 cells; however, HBsAg could be detected in the lysates of THP-1 cells that were incubated with two huE6F6 antibodies, and this effect was obviously dose dependent. However, the phagocytosis effect of huE6F6-fuc- was stronger than that of huE6F6-wt; the EC50 of huE6F6-fuc- was 403.2 ng/mL, while the EC50 of huE6F6-wt was 1071 ng/mL.

DyLight 488-labeled HBsAg mixed with antibodies was added to THP-1 cells to measure the phagocytosis of HBsAg by THP-1 cells via flow cytometry. As shown in Figures 5A, B group of fluorescent THP-1 cells were detectable due to the phagocytosis of HBsAg- DyLight 488 after the addition of the huE6F6 antibody. Compared with huE6F6-wt, the fluorescence intensity of the positive cells treated with huE6F6-fuc- was stronger, and the median fluorescence intensity of huE6F6-fuc- in the positive cells was 6337 MIF, while the median fluorescence intensity of huE6F6-wt in the positive cells was 3243 MIF. These results suggested that both huE6F6-wt and huE6F6-fuc- could mediate THP-1 cell phagocytosis of HBsAg, and the phagocytic efficiency mediated by huE6F6-fuc- was two times higher that of huE6F6-wt.

We used PBMCs from three healthy people as effector cells to measure the huE6F6-mediated phagocytic efficiency of natural human phagocytes, and the results are shown in Figures 5C,D. We found that for all three donors, huE6F6-fuc- could mediate a stronger opsonophagocytosis efficiency than huE6F6-wt (Figures 5C,D). The rate of huE6F6-fuc-mediated HBsAg (%) phagocytosis was 1.29 times that of huE6F6-wt-mediated HBsAg (%) phagocytosis for donor 1, 1.92 times that of huE6F6-wt-mediated HBsAg (%) phagocytosis for donor 2, and 2.23 times that of huE6F6-wt-mediated HBsAg (%) phagocytosis for donor 3. As shown in Figure 5D, the mean rate of huE6F6-wt-mediated phagocytosis was 19.96%, while the mean rate of huE6F6-fuc-mediated phagocytosis was 33.78%. That is, the efficiency huE6F6-fuc-mediated opsonophagocytosis is 1.69 times higher than that mediated by huE6F6-wt (*p* = 0.035). The opsonophagocytosis efficiency of the three donors was not the same, possibly because the immune status of the three donors was not the same, and the number of activated phagocytes was not consistent. Besides, allotypic variants of Fc-receptor on the surface of phagocytic immune cells in the sample may have impact on phagocytic efficiency. Studies have shown that CD16a has two distinct allotypes, F158 and V158, of which the less frequent V158 variant shows a higher affinity for IgG. Although neutrophils do not express CD16a on the surface, other immune cells with partial phagocytic functions also express CD16a receptors on the surface, such as macrophage cells.

In our previous study, we confirmed that the parent antibodies huE6F6 and E6F6 clear HBV through Fcγ receptor-mediated phagocytosis. The changes in the profiles of 32 cytokines in mouse serum after antibody injection were analyzed. The granulocyte colony-stimulating factor (G-CSF) level in the huE6F6-wt group and huE6F6-fuc- group showed a transient increase at 12 h after infusion (Figure 5E), but this level returned to baseline within 7 days (data not shown). The increase in G-CSF in the huE6F6-fuc- group was 175% of that in the huE6F6-wt group. This result suggests that stronger opsonophagocytosis occurred after treatment with huE6F6-fuc-. The improvement in the G-CSF level was most likely due to monocytes and macrophages activated by the antibody-antigen complexes. Then neutrophils may be one of the targets.

### 3.6 Enhanced opsonophagocytosis of huE6F6-fuc- is related to FcγRs

IgG-type antibodies trigger a series of immune responses by reacting with FcγR on the surface of immune cells. There are five main FcγRs in the human body: hCD64 (FcγR I), hCD32a (FcγR IIa), hCD32b (FcγR IIb), hCD16a (FcγR IIIa), and hCD16b (FcγR IIIb). hCD16b is mainly expressed on the surfaces of phagocytic cells (including neutrophils and macrophages). After the binding of the antibody Fc segment to hCD16b, phagocytes are activated to initiate phagocytosis. In this study, we tested the ability of huE6F6-wt and huE6F6-fuc- to bind to human and mouse FcγRs by CLA.

We found that the ability of the two antibody types to bind to hCD64 or hCD32a was basically the same, but the abilities of huE6F6-fuc- to bind to hCD32b, hCD16a or hCD16b were stronger than those of huE6F6-wt and the results are shown in Supplementary Table S3.

**FIGURE 6:**
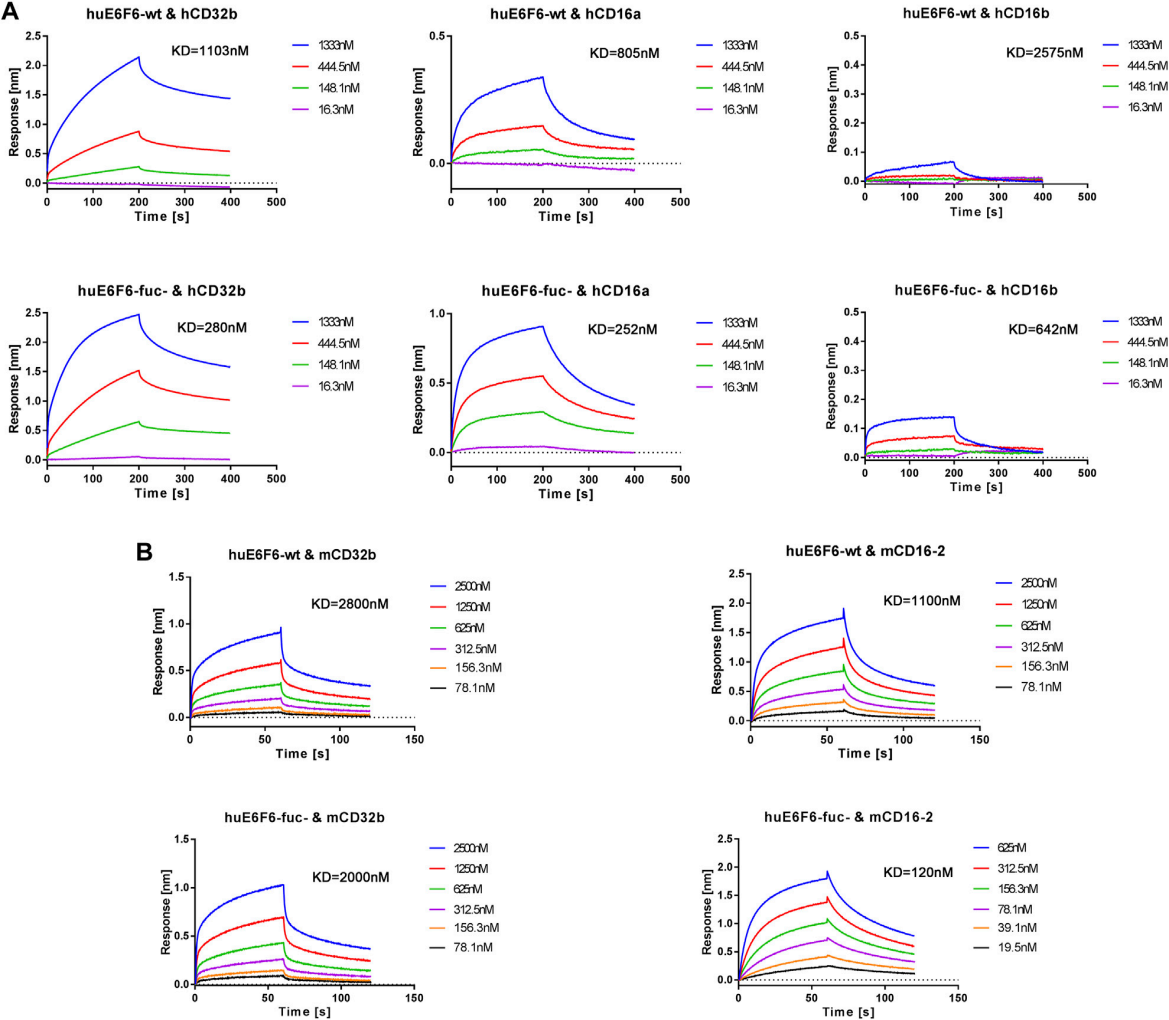
Representative BLI analysis of huE6F6-wt or huE6F6-fuc- binding to Fcγ receptors. **(A)** Kinetics of huE6F6-wt or huE6F6-fuc- binding to hCD32b, hCD16a, and hCD16b. **(B)** Kinetics of huE6F6-wt or huE6F6-fuc- binding to mCD32b and mCD16-2.

To further verify this hypothesis, we conducted affinity determination by BLI, and the results are shown in Figure 6A. We found that the affinity with which hCD32b bound to huE6F6-fuc was 3.9 times higher than that with which hCD32b bound to huE6F6-wt. The ability of huE6F6-fuc- to bind to hCD16a was 3.2 times higher than that of huE6F6-wt to bind to hCD16a. The affinity between hCD16b and huE6F6-fuc- was 4.0 times higher than that between hCD16b and huE6F6-wt. Therefore, the defucosylation of huE6F6 enhanced its ability to bind to huCD32b, huCD16a and huCD16b. This suggests that defucosylation of Fc may enhance antibody-mediated antigen phagocytosis.

It has been reported that the FcγRs of mice can be partially recognized by the Fc segment of human IgG. In this study, the abilities of huE6F6-wt or huE6F6-fuc- to bind to mouse FcγRs were also tested.

The binding of the two huE6F6 variants to mCD64 and mCD16 was basically consistent (data not shown), while the KD of mCD32b and huE6F6-fuc- was 2.00E-06 M and the KD of huE6F6-wt was 2.80E-06 M, which is a 1.4-fold increase in binding activity. The KD of mCD16-2 and huE6F6-fuc- was 1.20E-07 M, and the KD of mCD16-2 and huE6F6-wt was 1.10E-06 M, indicating a 9.2-fold increase in binding activity. mCD32b or mCD16-2 are mainly expressed on the surfaces of mouse phagocytic cells, and this receptor is related to phagocytosis. This suggests that human antibody-mediated phagocytosis can be evaluated in mice.

## 4 Discussion

The approval of an anti-HCV drug developed by Gilead has revolutionized the treatment of hepatitis C infection (Schreiber et al., 2016). This advance has increased scientists’ confidence that HCV will be eradicated worldwide by 2030 (Dore et al., 2020). However, there is a need to develop more effective treatments for hepatitis B infection. Antibody drugs have been successfully used in the treatment of many diseases. Glycoengineering is an effective way to improve the efficacy of antibody drugs, which has been proven in a variety of drugs, such as benralizumab (developed by MedImmune) (Laviolette et al., 2013) and mogamulizumab (developed by Kyowa Hakko Kirin) (Kanazawa et al., 2014; Remer et al., 2014). In addition to the field of antibody therapy for tumors, the glycosylation engineering technology has also been successfully used to achieve better therapeutic effects for antibodies in the field of infectious diseases, such as HIV, Dengue, RSV and SARS-CoV-2 (Oosterhoff, Janita J et al., 2022). Such glycoengineering was mostly done to increase effectors functions such as ADCC and/or to prevent ADE or antibody-dependent enhancement of infection.

In a previous study, we developed a novel therapeutic humanized antibody, namely, huE6F6, which can effectively suppress HBV in a disease model (Zhang et al., 2016; Zhou et al., 2020). In this study, we developed *FUT-8* and *GS-*knockout CHO cells and used them to express huE6F6 in order to generate huE6F6-fuc-. The characterization of its quality showed that huE6F6-fuc- has good homogeneity, low aggregation and degradation tendencies, and good thermal stability, which can support further development. In mouse models, more efficient HBsAg suppression was observed in the huE6F6-fuc- treatment group. We also explored the mechanism and confirmed that defucosylation of huE6F6 enhances immune clearance by enhancing the ability of the antibody Fc region to bind to certain Fc-gamma receptors.

Previous studies suggests that antibody mediated antigen clearance may be achieved through one or more mechanisms, including neutralization, ADCC, CDC, Fcγ-receptor-mediated opsonophagocytosis, and so on (Zhang et al., 2016). This means that enhancing one or more of these mechanisms could potentially enhance the antibody therapeutic effects. Neutralization is the most direct mechanism. After treatment with HIV-neutralizing antibodies, HIV viral clearance is mainly achieved through neutralization (Klein et al., 2012; Barouch et al., 2013). In this study, we found that huE6F6-fuc- had the same binding activity and *in vitro* neutralization activity as wild-type huE6F6. This suggests that defucosylation enhances antigen clearance but not by enhancing neutralization. Previous studies indicate that the size of the antibody-antigen complex correlates with phagocytic efficiency (Zhang et al., 2016; Bakalar et al., 2018). In 2016, it was revealed that E6F6, which is the parent of huE6F6, forms a smaller immune complex with HBsAg, which is possibly associated with its efficacy of opsonophagocytosis in viral clearance *in vivo* (Zhang et al., 2016). In this study, the size of the immune complex that was formed by antibodies and antigens was analyzed. The antibody‒antibody complex size was not changed by huE6F6 defucosylation (Supplementary Figure S5). This suggests that defucosylation of huE6F6 enhances viral clearance, independently of the change in the size of the antigen-antibody complex.

In studies about influenza and West Nile virus, it has been demonstrated that Fcγ receptor-dependent phagocytosis is a general pathway by which antibody-mediated viral clearance occurs (Huber et al., 2001; Chung et al., 2007; DiLillo et al., 2014). It is also one of the mechanisms underlying the therapeutic effects of E6F6, which is the parent molecule of the huE6F6 antibody. In this study, *in vitro* phagocytosis experiments also revealed that the defucosylation of huE6F6 enhanced the opsonophagocytosis of HBsAg. Analysis of serum cytokine levels in treated mice revealed a significant increase in G-CSF, which is most likely produced in monocytes and macrophages activated by the antibody-antigen complexes. Then neutrophils may be activated by the G-CSF.

In a previous study, we found that the main effector cells of E6F6 were Kupffer cells, F4/80 phagocytes and neutrophils (Zhang et al., 2016) but not NK cells. Kupffer cells, F4/80 phagocytes and neutrophils are all involved in phagocytosis. FcγRs on the surface of these three phagocytes bind to the Fc segment of E6F6 and mediate the immune clearance of ICs. Enhancing the binding of Fc and FcγRs may be an effective way to enhance immune clearance.

The binding between an antibody Fc region and Fc receptor has been proven to be related to the immune activity that is mediated by antibody. In the field of antibody drug development, Fc modification can be used to enhance the binding of antibodies and Fc receptors to enhance the therapeutic effect of antibodies. Conversely, the binding of antibodies to Fc receptors can also be reduced to improve the safety of antibody drugs (Saunders, 2019; Liu et al., 2020). In this research, it was found that defucosylation enhanced the binding of the huE6F6 antibody to hCD32b, hCD16a, hCD16b, mCD32 and mCD16-2, and these receptors are mainly located on the surface of phagocytic cells and are related to phagocytosis. This further confirms that defucosylation enhances the clearance of antigen/antibody complexes by enhancing binding to FcγR. However, hCD32b is homologous to mCD32, and hCD16a and hCD16b are homologous to mCD16-2 (Bruhns and Jonsson, 2015; Bournazos, 2019). In 2017, it was demonstrated that human IgG1 antibody can bind to mouse FcγR with a similar affinity (Dekkers et al., 2017). This suggests that human antibodies can be recognized by mouse FcγR and trigger corresponding immunophagocytosis, and these interactions are valuable for evaluating the human antibody-mediated immune clearance effect in mice. This means that in this research, transgenic mice, which were used as a model to study the potency of humanized antibodies, are valuable for predicting the therapeutic effects of future drugs in humans.

HCD16b is mainly expressed in neutrophils and some basophil cells, and it is inducibly expressed on eosinophils; all of these cells are related to the phagocytosis of antigen and antibody complexes (Bruhns and Jonsson, 2015; Roberts and Barb, 2018). hCD32b is expressed in B cells, DCs, basophils, mononuclear macrophages, and some neutrophils (Bruhns and Jonsson, 2015). huCD32b is also highly expressed on liver endothelial sinuoid cells and has been associated with the clearance of small immune complexes from the circulation by the liver (Bruhns and Jonsson, 2015). Both hCD32a and hCD32b can mediate the internalization of ICs, but the difference is that after endocytosis by hCD32a, the ICs are transferred to the lysosome, and the antibody and antigen are simultaneously cleared. hCD32b can mediate antibody recycling from the cell and maintain the half-life of antibodies. A previous study proved that the enhancing the binding of an antibody to hCD32a can result in platelet activation and aggregation, while enhancing the binding of an antibody to hCD32b did not cause similar adverse reactions (Mimoto et al., 2013). In this study, defucosylation only improved the binding of huE6F6 to hCD32b but did not change the binding of the antibody to hCD32a, which may enhance the endocytosis mediated by hCD32b or increase the effect of antigen presentation but will not increase the safety risk associated with the antibody. Defucosylation also increased the binding of other antibodies to CD16a, CD16b and CD32b, but the degree of improvement was not completely consistent (Subedi and Barb, 2016). This may occur because the differences in the variable region result in subtle structural changes in the constant region. Indeed, the increased affinity of decfucosylation for CD16a and CD16b receptors was observed in this study, and CD16a and CD16b are also expressed on human NK cells, so NK cells may be involved in ADCC and ADCP responses, which may be beneficial for the clearance of infected cells.

In the natural state, HBV virus could not infect wild type mice. In this study, HBV transgenic mice were used, which stably integrated the HBV1.2 ploidy genome in the chromosome of HBV transgenic mice, showing innate immune tolerance to HBV, and high levels of HBV DNA and HBsAg. The advantage of this model is that it is quite stable, and there was little difference in HBsAg level among mice, and the vast majority of bred mice are available for evaluation experiments, HBV carcinogenesis model can also be induced by low dose of DEN (Wu et al., 2010). This model has been used in a number of studies.

Our results may have broad implications in understanding the treatment mechanism of CHB passive immunotherapy. Taken together, the huE6F6-fuc- molecule that we generated is a superior candidate for the further development of therapeutic antibodies for use in CHB treatment. In this study, the half-life of antibodies in HBV-Tg mice was short, possibly due to the antigen accelerating antibody clearance. We predict that in humans, huE6F6-fuc- may have a longer half-life. Further modification of huE6F6-fuc-, such as modification to regulate pH dependence and antibody recirculation, may lead to a better clinical therapeutic effect.

## Data Availability

The data presented in the study are deposited in the NCBI SRA database repository, accession number PRJNA1050859

## References

[B1] Bakalar M. H. Joffe A. M. Schmid E. M. Son S. Podolski M. Fletcher D. A. (2018). Size-dependent segregation controls macrophage phagocytosis of antibody-opsonized targets. Cell 174, 131–142. 10.1016/j.cell.2018.05.059 29958103 PMC6067926

[B2] Barouch D. H. Whitney J. B. Moldt B. Klein F. Oliveira T. Y. Liu J. (2013). Therapeutic efficacy of potent neutralizing HIV-1-specific monoclonal antibodies in SHIV-infected rhesus monkeys. Nature 503, 224–228. 10.1038/nature12744 24172905 PMC4017780

[B3] Baum A. Ajithdoss D. Copin R. Zhou A. Lanza K. Negron N. (2020). REGN-COV2 antibodies prevent and treat SARS-CoV-2 infection in rhesus macaques and hamsters. Science 370, 1110–1115. 10.1126/science.abe2402 33037066 PMC7857396

[B4] Bournazos S. (2019). IgG Fc receptors: evolutionary considerations. Curr. Top. Microbiol. Immunol. 423, 1–11. 10.1007/82_2019_149 30739161

[B5] Bruhns P. Jonsson F. (2015). Mouse and human FcR effector functions. Immunol. Rev. 268, 25–51. 10.1111/imr.12350 26497511

[B6] Carter P. J. Lazar G. A. (2018). Next generation antibody drugs: pursuit of the 'high-hanging fruit. Nat. Rev. Drug Discov. 17, 197–223. 10.1038/nrd.2017.227 29192287

[B7] Chen C. L. Hsu J. C. Lin C. W. Wang C. H. Tsai M. H. Wu C. Y. (2017). Crystal structure of a homogeneous IgG-fc glycoform with the N-glycan designed to maximize the antibody dependent cellular cytotoxicity. ACS Chem. Biol. 12, 1335–1345. 10.1021/acschembio.7b00140 28318221

[B8] Chung K. M. Thompson B. S. Fremont D. H. Diamond M. S. (2007). Antibody recognition of cell surface-associated NS1 triggers Fc-gamma receptor-mediated phagocytosis and clearance of West Nile Virus-infected cells. J. virology 81, 9551–9555. 10.1128/JVI.00879-07 17582005 PMC1951387

[B10] Dekkers G. Bentlage A. E. H. Stegmann T. C. Heather L. H. Lissenberg-Thunnissen S. James Z. (2017). Affinity of human IgG subclasses to mouse Fc gamma receptors. mAbs 9, 767–773. 10.1080/19420862.2017.1323159 28463043 PMC5524164

[B11] Delidakis G. Kim J. E. George K. Georgiou G. (2022). Improving antibody therapeutics byManipulating the Fc domain: immunological and structural considerations. Annu. Rev. Biomed. Eng. 24, 249–274. 10.1146/annurev-bioeng-082721-024500 35363537 PMC9648538

[B12] Dienstag J. L. (2008). Hepatitis B virus infection. N. Engl. J. Med. 359, 1486–1500. 10.1056/NEJMra0801644 18832247

[B13] DiLillo D. J. Tan G. S. Palese P. Ravetch J. V. (2014). Broadly neutralizing hemagglutinin stalk-specific antibodies require FcγR interactions for protection against influenza virus *in vivo* . Nat. Med. 20, 143–151. 10.1038/nm.3443 24412922 PMC3966466

[B14] Dore G. J. Martinello M. Alavi M. Grebely J. (2020). Global elimination of hepatitis C virus by 2030: why not? Nat. Med. 26, 157–160. 10.1038/s41591-019-0706-x 32047317

[B15] European Association For The Study Of The L (2012). EASL clinical practice guidelines: management of chronic hepatitis B virus infection. J. hepatology 57, 167–185. 10.1016/j.jhep.2012.02.010 22436845

[B16] Fanning G. C. Zoulim F. Hou J. Bertoletti A. (2019). Therapeutic strategies for hepatitis B virus infection: towards a cure. Nat. Rev. Drug Discov. 18, 827–844. 10.1038/s41573-019-0037-0 31455905

[B17] Goletz C. Lischke T. Harnack U. Schiele P. Danielczyk A. Rühmann J. (2018). Glyco-engineered anti-human programmed death-ligand 1 antibody mediates stronger CD8 T cell activation than its normal glycosylated and non-glycosylated counterparts. Front. Immunol. 9, 1614. 10.3389/fimmu.2018.01614 30061887 PMC6054930

[B18] Hou J. L. Zhao W. Lee C. Hann H. W. Peng C. Y. Tanwandee T. (2020). Outcomes of long-term treatment of chronic HBV infection with entecavir or other agents from a randomized trial in 24 countries. Clin. Gastroenterol. Hepatol. 18, 457–467. 10.1016/j.cgh.2019.07.010 31306800

[B19] Huber V. C. Lynch J. M. Bucher D. J. Le J. Metzger D. W. (2001). Fc receptor-mediated phagocytosis makes a significant contribution to clearance of influenza virus infections. J. Immunol. 166, 7381–7388. 10.4049/jimmunol.166.12.7381 11390489

[B20] Jeng W. J. Papatheodoridis G. V. &Lok A. S. F. (2023). Hepatitis B. Lancet 401, 1039–1052. 10.1016/S0140-6736(22)01468-4 36774930

[B21] Kanazawa T. Hiramatsu Y. Iwata S. Siddiquey M. Sato Y. Suzuki M. (2014). Anti-CCR4 monoclonal antibody mogamulizumab for the treatment of EBV-associated T- and NK-cell lymphoproliferative diseases. Clin. cancer Res. official J. Am. Assoc. Cancer Res. 20, 5075–5084. 10.1158/1078-0432.CCR-14-0580 25117294

[B22] Klein F. Halper-Stromberg A. Horwitz J. A. Gruell H. Scheid J. F. Bournazos S. (2012). HIV therapy by a combination of broadly neutralizing antibodies in humanized mice. Nature 492, 118–122. 10.1038/nature11604 23103874 PMC3809838

[B23] Kremsdorf D. Soussan P. Paterlini-Brechot P. Brechot C. (2006). Hepatitis B virus-related hepatocellular carcinoma: paradigms for viral-related human carcinogenesis. Oncogene 25, 3823–3833. 10.1038/sj.onc.1209559 16799624

[B24] Kwon H. Lok A. S. (2011). Hepatitis B therapy. Nat. Rev. Gastroenterol. Hepatol. 8, 275–284. 10.1038/nrgastro.2011.33 21423260

[B25] Laviolette M. Gossage D. L. Gauvreau G. Leigh R. Olivenstein R. Katial R. (2013). Effects of benralizumab on airway eosinophils in asthmatic patients with sputum eosinophilia. J. allergy Clin. Immunol. 132, 1086–1096. 10.1016/j.jaci.2013.05.020 23866823 PMC4172321

[B26] Li D. He W. Liu X. Zheng S. Qi Y. Li H. (2017). A potent human neutralizing antibody Fc-dependently reduces established HBV infections. Elife 6, e26738. 10.7554/eLife.26738 28949917 PMC5614562

[B27] Liu R. Oldham R. J. Teal E. Beers S. A. Cragg M. S. (2020). Fc-engineering for modulated effector functions-improving antibodies for cancer treatment. Antibodies (Basel) 9, 64. 10.3390/antib9040064 33212886 PMC7709126

[B28] Marasco W. A. Sui J. (2007). The growth and potential of human antiviral monoclonal antibody therapeutics. Nat. Biotechnol. 25, 1421–1434. 10.1038/nbt1363 18066039 PMC7097443

[B29] Mimoto F. Katada H. Kadono S. Igawa T. Kuramochi T. Muraoka M. (2013). Engineered antibody Fc variant with selectively enhanced FcγRIIb binding over both FcγRIIa(R131) and FcγRIIa(H131). Protein Eng. Des. Sel. PEDS 26, 589–598. 10.1093/protein/gzt022 23744091 PMC3785249

[B30] Nayagam S. Thursz M. Sicuri E. Conteh L. Wiktor S. Low-Beer D. (2016). Requirements for global elimination of hepatitis B: a modelling study. Infect. Dis. 16, 1399–1408. 10.1016/S1473-3099(16)30204-3 27638356

[B31] Nimmerjahn F. Ravetch J. V. (2008). Fcgamma receptors as regulators of immune responses. Nat. Rev. Immunol. 8, 34–47. 10.1038/nri2206 18064051

[B32] Organization W. H. (2021). World health organization fact sheet (revised 27 july 2021). Hepatitis B.

[B33] Rajendra Y. Hougland M. D. Alam R. Morehead T. A. Barnard G. C. (2015). A high cell density transient transfection system for therapeutic protein expression based on a CHO GS-knockout cell line: process development and product quality assessment. Biotechnol. Bioeng. 112 (5), 977–986. 10.1002/bit.25514 25502369

[B34] Regeneron (2021). Fda authorizes lower 1,200 MG intravenous and subcutaneous dose of REGEN-COV™ (casirivimab and imdevimab) antibody cocktail to treat patients with COVID-19. TARRYTOWN, N.Y.

[B35] Remer M. Al-Shamkhani A. Glennie M. Johnson P. (2014). Mogamulizumab and the treatment of CCR4-positive T-cell lymphomas. Immunotherapy 6, 1187–1206. 10.2217/imt.14.94 25496334

[B36] Resch B. (2017). Product review on the monoclonal antibody palivizumab for prevention of respiratory syncytial virus infection. Hum. vaccines Immunother. 13, 2138–2149. 10.1080/21645515.2017.1337614 PMC5612471 28605249

[B37] Roberts J. T. Barb A. W. (2018). A single amino acid distorts the Fc gamma receptor IIIb/CD16b structure upon binding immunoglobulin G1 and reduces affinity relative to CD16a. J. Biol. Chem. 293, 19899–19908. 10.1074/jbc.RA118.005273 30361439 PMC6314127

[B38] Saunders K. O. (2019). Conceptual approaches to modulating antibody effector functions and circulation half-life. Front. Immunol. 10, 1296. 10.3389/fimmu.2019.01296 31231397 PMC6568213

[B39] Schreiber J. McNally J. Chodavarapu K. Svarovskaia E. Moreno C. (2016). Treatment of a patient with genotype 7 hepatitis C virus infection with sofosbuvir and velpatasvir. Hepatology 64, 983–985. 10.1002/hep.28636 27177605

[B40] Shen C. Zhang M. Chen Y. Zhang L. Wang G. Chen J. (2019). An IgM antibody targeting the receptor binding site of influenza B blocks viral infection with great breadth and potency. Theranostics 9, 210–231. 10.7150/thno.28434 30662563 PMC6332795

[B41] Shen Z. Wu J. Gao Z. Wang J. Zhu H. Mao R. (2020). Characterization of IL-21-expressing recombinant hepatitis B virus (HBV) as a therapeutic agent targeting persisting HBV infection. Theranostics 10, 5600–5612. 10.7150/thno.44715 32373234 PMC7196313

[B42] Subedi G. P. Barb A. W. (2016). The immunoglobulin G1N-glycan composition affects binding to each low affinity Fc γ receptor. mAbs 8, 1512–1524. 10.1080/19420862.2016.1218586 27492264 PMC5098437

[B43] Tang P. K. (2017). Palivizumab prophylaxis in preterm infants. Lancet Respir. Med. 5, 171. 10.1016/S2213-2600(17)30050-4 28219614

[B44] Trepo C. Chan H. L. Lok A. (2014). Hepatitis B virus infection. Lancet 384, 2053–2063. 10.1016/S0140-6736(14)60220-8 24954675

[B45] Wada R. Matsui M. Kawasaki N. (2018). Influence of N-glycosylation on effector functions and thermal stability of glycoengineered IgG1 monoclonal antibody with homogeneous glycoforms. mAbs 11, 350–372. 10.1080/19420862.2018.1551044 30466347 PMC6380427

[B46] Wang W. Sun L. Li T. Ma Y. Li J. Liu Y. (2016). A human monoclonal antibody against small envelope protein of hepatitis B virus with potent neutralization effect. mAbs 8, 468–477. 10.1080/19420862.2015.1134409 26713590 PMC4966830

[B47] Wang Y. Mei Y. Ao Z. Chen Y. Jiang Y. Chen X. (2022). A broad-spectrum nanobody targeting the C-terminus of the hepatitis B surface antigen for chronic hepatitis B infection therapy. Antivir. Res. 199, 105265. 10.1016/j.antiviral.2022.105265 35183645

[B48] Weinreich D. M. Sivapalasingam S. Norton T. Ali S. Gao H. Bhore R. (2021). REGN-COV2, a neutralizing antibody cocktail, in outpatients with covid-19. N. Engl. J. Med. 384, 238–251. 10.1056/NEJMoa2035002 33332778 PMC7781102

[B49] Wong S. K. Li A. Lanctot K. L. Paes B. (2018). Adherence and outcomes: a systematic review of palivizumab utilization. Expert Rev. Respir. Med. 12, 27–42. 10.1080/17476348.2018.1401926 29130355

[B50] Wu M. H. Ma W. L. Hsu C. L. Chen Y. L. Ou J. H. Ryan C. K. (2010). Androgen receptor promotes hepatitis B virus-induced hepatocarcinogenesis through modulation of hepatitis B virus RNA transcription. Sci. Transl. Med. 2 (32), 32ra35. 10.1126/scitranslmed.3001143 PMC3032595 20484730

[B51] Wu S. Zeng N. Sun F. Zhou J. Wu X. Sun Y. (2021). Hepatocellular carcinoma prediction models in chronic hepatitis B: a systematic review of 14 models and external validation. Clin. Gastroenterol. Hepatol. 19, 2499–2513. 10.1016/j.cgh.2021.02.040 33667678

[B52] Yamane-Ohnuki N. Kinoshita S. Inoue-Urakubo M. Kusunoki M. Iida S. Nakano R. (2004). Establishment of FUT8 knockout Chinese hamster ovary cells: an ideal host cell line for producing completely defucosylated antibodies with enhanced antibody-dependent cellular cytotoxicity. Biotechnol. Bioeng. 87, 614–622. 10.1002/bit.20151 15352059

[B53] You M. Liu Y. Chen Y. Guo J. Wu J. Fu Y. (2013). Maximizing antibody production in suspension-cultured mammalian cells by the customized transient gene expression method. Biosci. Biotechnol. Biochem. 77, 1207–1213. 10.1271/bbb.120968 23748758

[B54] You M. Yang Y. Zhong C. Chen F. Wang X. Jia T. (2018). Efficient mAb production in CHO cells with optimized signal peptide, codon, and UTR. Appl. Microbiol. Biotechnol. 102, 5953–5964. 10.1007/s00253-018-8986-5 29740673

[B55] Zhang J. F. Xiong H. L. Cao J. L. Wang S. J. Guo X. R. Lin B. Y. (2018). A cell-penetrating whole molecule antibody targeting intracellular HBx suppresses hepatitis B virus via TRIM21-dependent pathway. Theranostics 8, 549–562. 10.7150/thno.20047 29290826 PMC5743566

[B56] Zhang T. Y. Guo X. R. Wu Y. T. Kang X. Z. Zheng Q. B. Qi R. Y. (2020). A unique B cell epitope-based particulate vaccine shows effective suppression of hepatitis B surface antigen in mice. Gut 69, 343–354. 10.1136/gutjnl-2018-317725 30926653 PMC6984059

[B57] Zhang T. Y. Yuan Q. Zhao J. H. Zhang Y. L. Yuan L. Z. Lan Y. (2016). Prolonged suppression of HBV in mice by a novel antibody that targets a unique epitope on hepatitis B surface antigen. Gut 65, 658–671. 10.1136/gutjnl-2014-308964 26423112

[B58] Zhou B. Xia L. Zhang T. You M. Huang Y. He M. (2020). Structure guided maturation of a novel humanized anti-HBV antibody and its preclinical development. Antivir. Res. 180, 104757. 10.1016/j.antiviral.2020.104757 32171857

[B59] Zoulim F. (2007). Emerging drugs for hepatitis B. Expert Opin. Emerg. Drugs 12, 199–217. 10.1517/14728214.12.2.199 17604497 PMC3194402

