# Supplementary material for: A glycoengineered therapeutic anti-HBV antibody that allows increased HBsAg immunoclearance improves HBV suppression in vivo
Source: Front Pharmacol. 2023 Dec 27;14:1213726. doi: 10.3389/fphar.2023.1213726 (PMC10777313; doi:10.3389/fphar.2023.1213726)
Supplement: Supplementary file 1 [file DataSheet1.PDF]

Supplementary Material

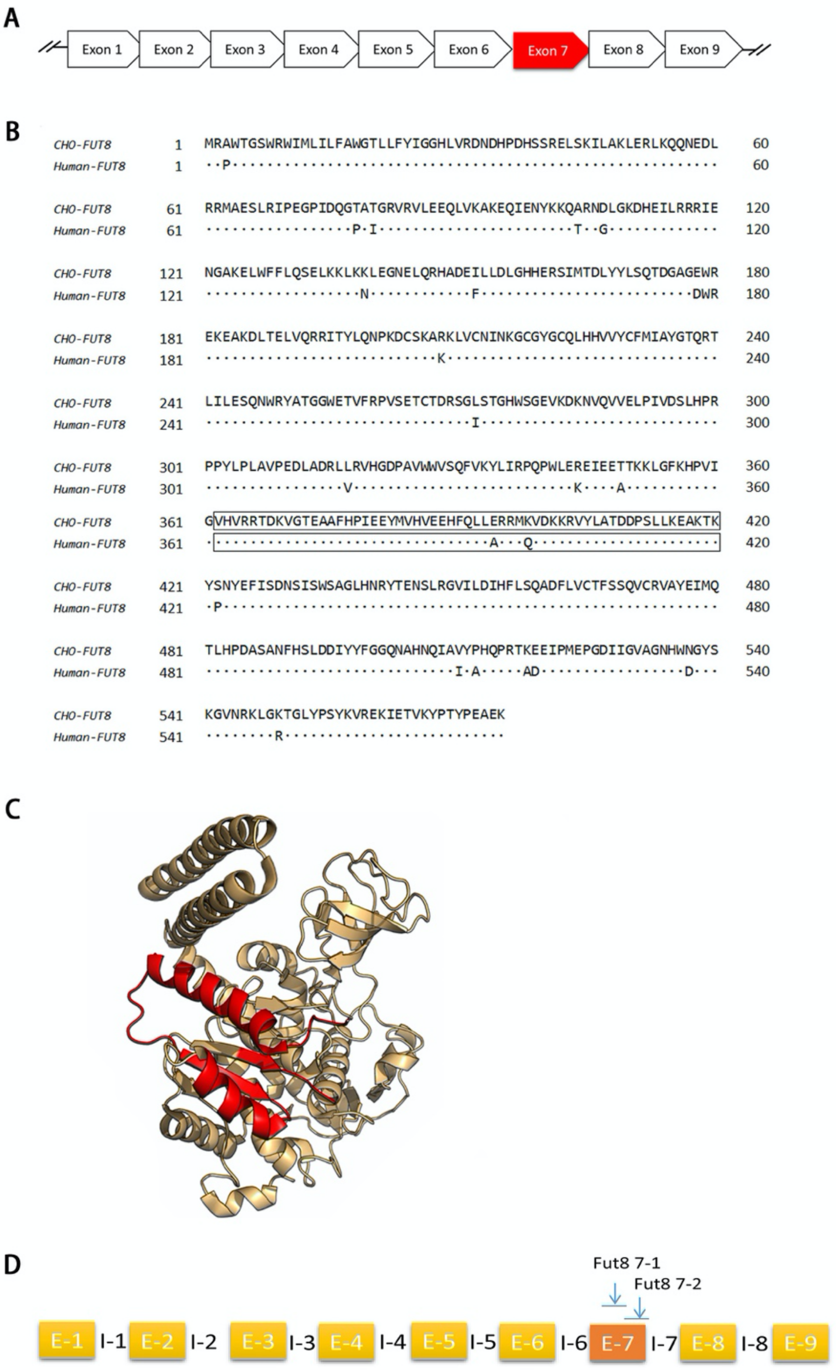

Fig. S1. The design of *FUT8* knocking out.

(A) Structure of CHO *FUT8* gene, exon 7(in red) is the catalytic core of the enzyme.

(B) The CHO *FUT8* gene and human *FUT8* were aligned. the same amino acid was substituted by *, and the region boxed was the exon 7.

(C)Crystal structure of human Fut8 protein (PDB:2DE0), the exon 7 is in red.

(D)The location of sgRNAs recognition is shown.

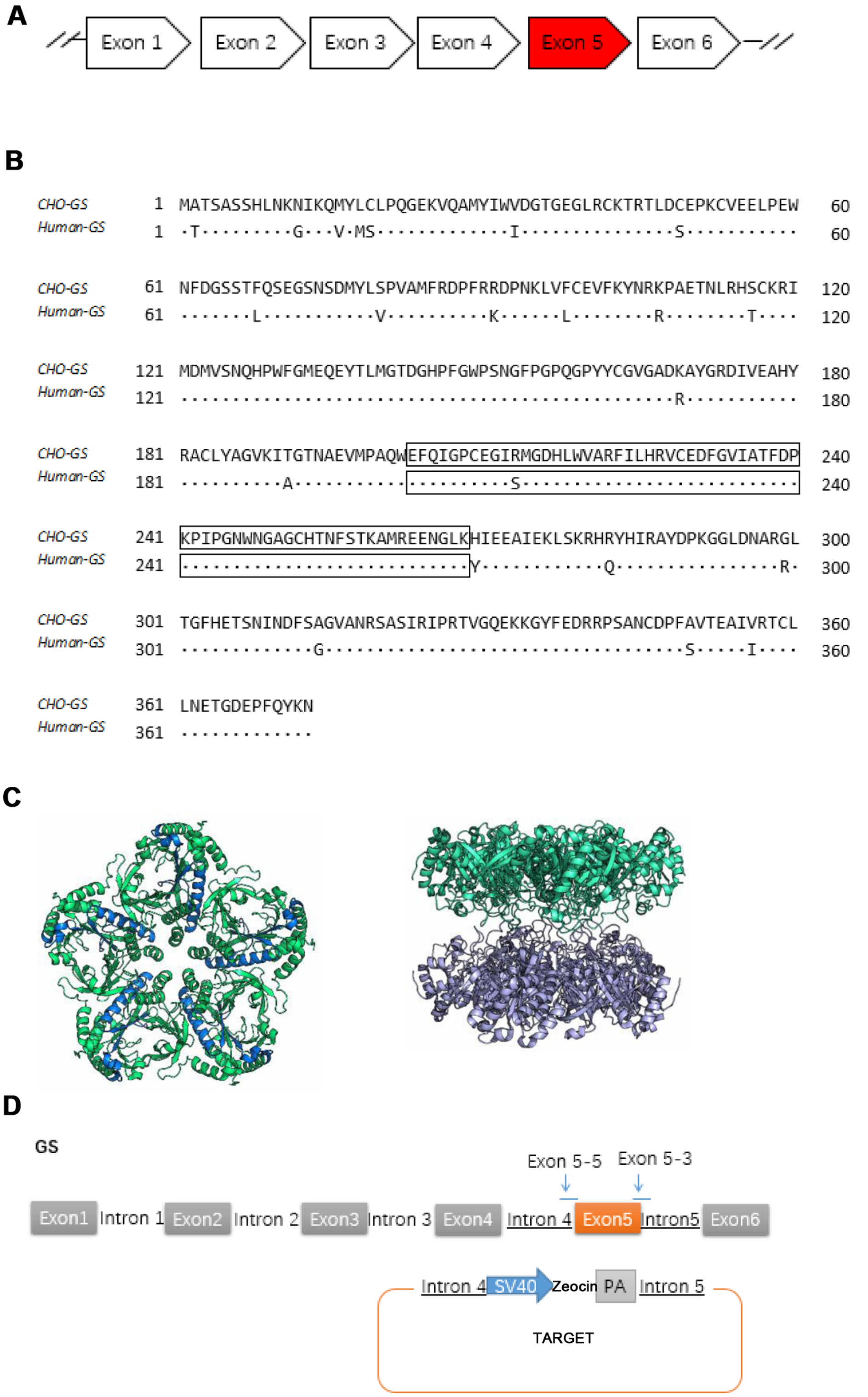

Fig. S2. The design of *GS* knocking out.

(A) Structure of CHO *GS* gene, exon 5(in red) is located on polymeric interface.
(B) The CHO *GS* gene and human *GS* were aligned. the same amino acid was substituted by *, and the region boxed was the exon 5.
(C) Crystal structure of human Fut8 protein (PDB:2QC8), the exon 5 is in blue.
(D) The location of sgRNAs and the design of homologous recombination.

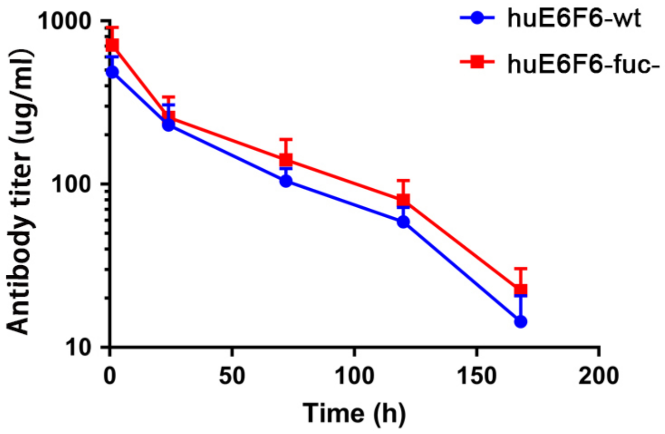

Fig. S3. In vivo Pk and PD profiles of huE6F6 in mouse.

huE6F6-wt and huE6F6-Fuc- were injected into HBV-Tg mice at a dose of 20mg/kg. Serum samples were collected at different time points and concentrations were determined uniformly.

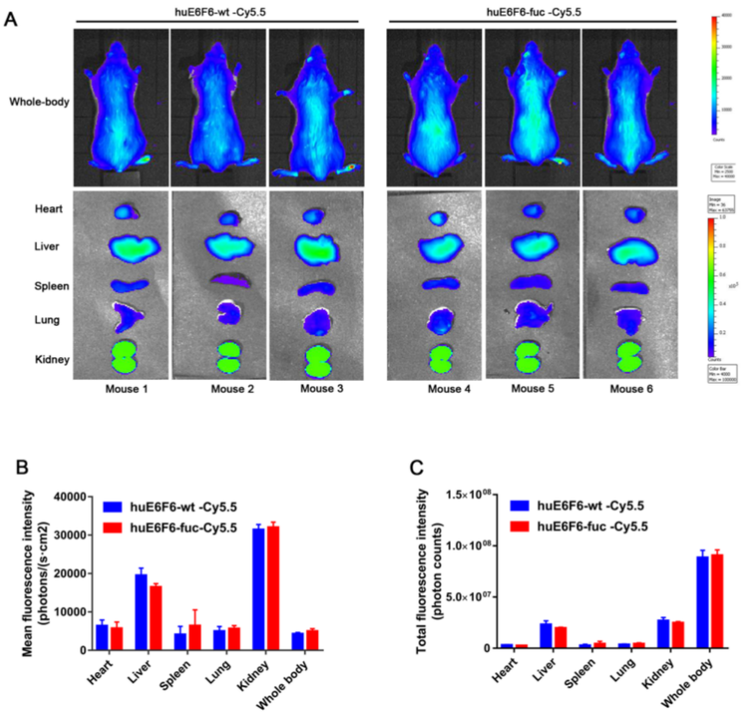

Fig. S4. In vivo tracking of huE6F6-wt and huE6F6-fuc- antibody distributions with near-infrared fluorescent dyes in mice.

Cy5.5 labeled huE6F6-wt and huE6F6-fuc- (5 mg/kg) were injected into BALB/c mice. (A) Fluorescence images of whole animals and isolated tissues harvested at 24 h after mAb infusions. Semi-quantitative analyses of (A) using the software package included with the *in vivo* imaging system on total (B) and mean (C) fluorescence intensity.

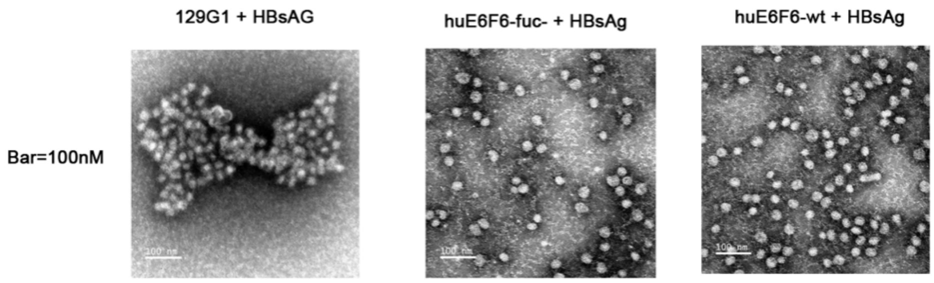

Fig. S5. Characterization of the mAb-Antigen immune complex by TEM. 129G1 is an antibody targeted to an epitope on HBsAg different from E6F6. It was found that 129G1 triggers HBsAg aggregation, because of the formation of cross-linked immune complexes. huE6F6-wt or huE6F6-fuc- forms a smaller antibody–antigen immune complex when they bind to antigen.

Tab.S1. The Tm of huE6F6-wt and huE6F6-fuc-

	Tm1 (°C)	Tm2 (°C)
**huE6F6-wt-1**	71.58	81.51
**huE6F6-wt-2**	71.60	81.47
**huE6F6-wt-3**	71.20	81.32
**huE6F6-fuc-1**	71.80	80.99
**huE6F6-fuc-2**	71.63	81.12
**huE6F6-fuc-3**	71.52	81.01

Tab.S2. The pK/PD parameters of huE6F6-wt and huE6F6-fuc-

**PK Paremeters**		**AUC (0-t)**	**AUC (0-∞)**	**MRT (0-t)**	**MRT (0-∞)**	**t1/2z**	**Tlast**	**CLz**	**Vz**	**C0**
**Unit**		**ug/L*h**	**ug/L*h**	**h**	**h**	**h**	**h**	**L/h/kg**	**L/kg**	**ug/L**
huE6F6-wt	No1	19,004,770	19,335,801	39.545	42.389	27.846	168	0.001	0.042	517,047.07
	No2	16,723,498	17,135,449	44.318	48.202	30.268	168	0.001	0.051	415,739.25
	No3	25,916,148	27,044,397	39.315	46.427	34.176	168	0.001	0.036	666,708.02
	No4	26,295,146	29,382,891	48.252	55.893	53.026	168	0.001	0.052	359,801.05
	No5	24,092,872	24,671,205	42.164	46.151	31.817	168	0.001	0.037	473,600.4
	**Mean**	**22,406,487**	**23,513,949**	**42.719**	**47.812**	**35.427**	**168**	**0.001**	**0.044**	**486,579.16**
	SD	4,305,558.2	5,157,276	3.713	4.989	10.105	0	0	0.008	116,869.73
huE6F6-fuc-	No1	20,555,508	21,094,539	36.301	41.108	35.447	168	0.001	0.048	647,161.57
	No2	25,640,074	26,497,844	44.032	49.591	32.449	168	0.001	0.035	652,162.89
	No3	32,590,598	33,869,051	46.268	52.783	34.184	168	0.001	0.029	772,727.73
	No4	29,358,302	31,457,977	55.632	66.502	42.9	168	0.001	0.039	469,987.94
	No5	37,247,511	38,468,796	34.812	40.581	35.357	168	0.001	0.027	1,019,030.5
	**Mean**	**29,078,399**	**30,277,641**	**43.409**	**50.113**	**36.067**	**168**	**0.001**	**0.036**	**712,214.12**
	SD	6,395,506.8	6,708,253.6	8.402	10.582	4.007	0	0	0.008	202,675.66

Tab.S3. The parameters of huE6F6-wt and huE6F6-fuc- binding to the Fcγ reccеptors

Ligand	Analyte	KD (M)	RMax	R^2	Fit Model	Improve fold
mCD32b-His	huE6F6-wt	2.80E-06	1.7339	0.9997	Steady State	1.4
	huE6F6-fuc-	2.00E-06	1.634	0.9973		
mCD16-2-His	huE6F6-wt	1.10E-06	2.2868	0.9975		9.2
	huE6F6-fuc-	1.20E-07	2.0198	0.996		
hCD32b-His	huE6F6-wt	1.103E-06	3.4955	0.9998		3.9
	huE6F6-fuc-	2.798E-07	2.6072	0.9938		
hCD16a- His	huE6F6-wt	8.047E-07	0.4877	0.8923		3.2
	huE6F6-fuc-	2.528E-07	0.9732	0.9651		
hCD16b- His	huE6F6-wt	2.575E-06	0.1857	0.9756		4.0
	huE6F6-fuc-	6.420E-07	0.1991	0.9385		
